# Development and evaluation of prevention bundle for neonatal healthcare-associated infections: an interventional study

**DOI:** 10.12688/f1000research.132819.2

**Published:** 2024-06-03

**Authors:** Usha Rani, Leslie E Lewis, Kiran Chawla, Anup Naha, Praveen Kumar

**Affiliations:** 1 Dept. of Social and Health Innovation, Prasanna School of Public Health (PSPH), Manipal Academy of Higher Education, Manipal, Karnataka, 576104, India; 2 Department of Pediatrics, Kasturba Medical College, Manipal, Manipal Academy of Higher Education, Manipal, Karnataka, 576104, India; 3 Department of Microbiology, Kasturba Medical College, Manipal, Manipal Academy of Higher Education, Manipal, Karnataka, 576104, India; 4 Department of Pharmaceutics, Manipal College of Pharmaceutical Sciences, Manipal Academy of Higher Education, Manipal, Karnataka, 576104, India; 5 Dept. of Commerce, Manipal Academy of Higher Education, Manipal, Karnataka, 576104, India

**Keywords:** healthcare-associated infections, mixed-method, nosocomial, prevention, neonate

## Abstract

**Background:**

Neonatal healthcare-associated infection (HAI) globally is the leading preventable cause of neonatal mortality. Neonatal mortality in India is also very high. Considering that HAIs can be prevented globally, there are disparities in resources; the current study aimed at developing locally feasible and effective prevention bundles for neonatal HAIs.

**Methods:**

A mixed-method study was conducted at one tertiary care teaching hospital's level IV Neonatal Intensive Care Unit. The study explores the causes of neonatal HAIs, current processes, benchmark practices, gaps in current practices with HAIs, root-cause analysis and system process mapping, and failure mode effect analysis. Observations, interviews, brainstorming activities, and a survey were conducted. Written and audio-video recorded prevention bundle was developed and implemented using a quasi-experimental study design.

**Results:**

Process standardisation, healthcare worker training, hand hygiene practices, nursing care process and vascular access process were identified as key improvement areas to prevent neonatal HAIs. Out of eighteen identified processes, three processes were standardised. All the healthcare providers were trained at three-time intervals of three months each. After implementing the prevention bundle, there was a significant decline in the rate of HAIs, reducing it from 9.6 to 7.0 per 100 admissions >48 hours. The bacteraemia rate fell from 5.2 per 1000 patient days to 2.6 per 1000 patient days and was statistically significant on a two-tailed student t-test with 95% CI with p-value=0.00073.

**Conclusions:**

Our developed prevention bundle for neonatal HAIs was significantly effective and reproducible for healthcare workers' training and development. Considering variations in global infection control practices and resources constraint, it is effective to develop a local prevention bundle for neonatal HAIs.

## Introduction

Globally 2.5 million neonatal mortalities were recorded in 2017, accounting for 47% of all under-five mortality that has increased by 7% in 27 years. ^1^ More than 1/3 ^rd^ of neonatal mortality is due to preventable neonatal infections. ^2^

Attention has been paid to reduce the neonatal mortality rate to the minimum however, many countries have failed to do so despite putting in a lot of effort. Efforts have been made not only from each country but also from the United Nations Children’s Fund (UNICEF), the World Health Organization (WHO) and various other non-profit organizations. The average neonatal mortality rate has reduced from 85.2 in 1969 to 22.7 in the year 2018 where the lowest motility rate in 1969 was 78.7 and the highest was 92.2 in contrast to 2018 it was 20.1 and 35.3 respectively. ^3^ The neonatal mortality rate is expressed per thousand live birth within the first 28 days of life. As per the country-wise estimation, the lowest neonatal mortality rate reported in 2018 was 0.9 in Japan and San Marino, and the highest was 42 in Pakistan per thousand live births around the globe. ^3^

The literature reports high neonatal healthcare-associated infections (HAIs) in India that range from 8% to 36.3%, however, the unreported number may differ from the reported. ^4^^–^^7^ The HAIs can contribute up to 30% mortality that can be prevented if timely appropriate measures are adopted. ^8^

A prevention bundle is a structure of three to five systematic activities of care developed scientifically, that when implemented together should bring down the rate of HAIs. ^9^ The concept of ‘bundles’ is developed by the Institute of Healthcare Improvements to help the healthcare providers, so they can deliver the best possible care to patients undergoing treatment with high risk. ^10^^,^^11^ Development of prevention bundle involves science focusing on the method of execution as it describes how to deliver the best care. A bundle is a package of tools improving habits and critical process with clear parameters. ^10^^,^^12^

According to a publication in 2013 by the International Nosocomial Infection Control Consortium, development, implementation and adherence to neonatal HAIs prevention bundle can reduce up to 54% HAIs and up to 58% reduction in related mortality. ^13^ Healthcare-associated infections (HAIs) are always a threat to healthcare providers and the patient. Various preventive measures are proposed by many researchers around the globe to prevent HAIs. The neonatal population is most vulnerable and susceptible to get HAIs. The majority of preventable practices are reported, tried, and tested in developed countries on the adult population and limited among neonates.

A report by the World Health Organization (WHO) published in 2011 reported that the surveillance system for healthcare-associated infections in high-income countries is capturing the rate of HAIs but its existence in low and middle-income countries is scanty. ^14^^,^^15^

*Preventive measures for neonatal HAIs:* A prevention bundle developed by ‘International Nosocomial Infection Control Consortium’ for Ventilator Associated Pneumoniae (VAP) in the neonatal population has been reported in 2012, however, no prevention bundle for other types of infections has been reported for the neonatal population. ^16^ Since the increase in the amount of published literature related to the prevention of HAIs is increasing, it becomes difficult for a healthcare provider to decide which practice to follow in neonatal intensive care unit considering the limited availability of resources. Neonatal mortality in India is primarily due to intrapartum related complications, sepsis, meningitis, pneumonia, congenital abnormalities, and other neonatal disorders in chronological order. ^8^

A healthcare worker can try to practice cluster care for maximum possible invasive and non-invasive procedures that might help to reduce the contact frequency with neonate. ^17^ Clustered care can be practised for delivery of medication, withdrawal of blood sample for investigation, providing routine nursing care to the neonate and any suctioning if required. Supervision and surveillance while securing intravenous line, preparation and delivery of medications might also be beneficial to reduce bloodstream infections. ^18^

As per the guidelines of Centers for Disease Control and Prevention (CDC) and WHO active surveillance is required to identify the source of infection. Various preventive measures are proposed by many researchers around the globe to prevent HAIs. ^14^^,^^19^ The neonatal population is most vulnerable and susceptible to getting HAIs. Compared with adults and paediatrics, neonates are more vulnerable to acquiring infection. The prevention bundle is available for the adult population and modification of the same prevention bundle is practised for Paediatrics and neonates in some countries.

*Care of Intravenous (IV) Lines: Blood Stream Infection:* The prevention of BSI involves bundled approaches as hand hygiene, scrubbing the outer surface of catheter hub by using 2% chlorhexidine in 70% isopropyl alcohol, use of standard precautions, changing the catheter dressing if soiled. ^20^

*Central Line-Associated Blood Stream Infections (CLABSI):*The prevention of CLABSI is by following aseptic techniques while inserting central venous catheters, avoiding the femoral site and removing the unnecessary catheters which are present. ^21^ Skin preparation using all aseptic measures and daily assessment of the catheter site is vital. ^22^ Central lines should be removed as early as possible if they are not needed. ^23^^,^^24^

*Catheter-related bloodstream infections (CRBSI):* In the case of neonates the prevention of CRBSI is by various practices such as hand hygiene before and after patient contact, using gloves for all invasive procedures, following standardized procedures, educating the HCWs regarding infection control measures, ^25^^,^^26^ surveillance of NICU, disinfecting the site of insertion, and strict aseptic precautions are taken while inserting the catheter. ^21^ A sterile gauze is covered over the three-way stopcocks and should be accessed by using sterile gloves ^27^ but the use of a three-way stopcock is not much appreciated now. All vascular hubs needleless connectors and injection ports must be disinfected using either 70% alcohol or 4% chlorhexidine solution before the access can help in the reduction of BSI, ^28^ catheter hub care should be demonstrated and practised regularly and the catheter dressing should be changed when it is soiled. ^29^

*Development of guidelines:* Standardized guidelines are developed for the infection control practices by which the infection rates are reduced for neonates. These practices include handwashing, infant handling, use of gloves, care of intravenous lines, handling of three-way-stopcock and endotracheal tube suctioning.

Considering the limitation of the information, the economy of a country and available resources implementing preventable measures becomes a challenge. There are various infection control practices with evidence reported. It is the hospital infection control team that decides which practice to follow in their respective ICU. There is variability in thinking, approach, limiting resources and various other factors, no two-healthcare facility can adapt similar prevention approach. The majority of preventable practices are reported, tried, and tested in developed countries on the adult population and limited among neonates. ^30^^–^^32^ There is an increase in the number of published literature related to the prevention of HAIs in the adult population, it becomes difficult for a healthcare provider to decide which practice to follow in neonatal intensive care unit considering the limited availability of resources.

There is a dearth of literature from developing nations on the adoption of available solutions to practice and care bundles to prevent neonatal HAIs. Implementation of care bundles adopted by developed nations to prevent HAIs adds costs to healthcare and the patient. Identifying the domestic concerns causing neonatal HAIs and finding solutions to prevent neonatal HAIs with minimal cost burden needs further research.

Research using NICU system process mapping to explore the potential contributors and eliminate them to prevent HAIs is not explored. There is a scarcity of scientific research on standardizing the process for vascular access and maintenance, nursing care and emergency handling targeting to prevent neonatal HAIs while delivering the standard of care. Identifying and standardizing the potential critical steps of care processes to prevent neonatal HAIs might help healthcare professionals upgrade their practices while delivering care to neonates and reduce the domestic rates of HAIs. The current study was carried out to develop and evaluate prevention bundles for neonatal healthcare-associated infections (HAIs).

## Methods

### Study design

This is a mixed method study combining the results obtained through qualitative and quantitative studies. The study was carried out in a level IV Neonatal Intensive Care Unit (NICU) at a tertiary care teaching hospital located in coastal Karnataka, India from December 2016 to July 2020. To develop the prevention bundle, we needed to identify the factors contributing to neonatal HAIs ^33^; we did root cause analysis, ^34^ identifying the failure mode and effect analysis ^35^^,^^36^ followed by system process mapping. ^37^ Once we identified the system and its process contributing to HAIs, we observed in other benchmark hospitals ^38^ the policy, practices and process to prevent neonates from HAIs also carried out a review on the prevention practices. The prevention bundle was developed as the standard processes ^39^^–^^41^ and to implement the bundles we needed to train the healthcare provider providing direct care to the neonate. Audio-video recording, process flow, various levels of workstations as training method to deliver the behaviour and practice change was selected. Pre and Post training knowledge assessment was also carried out using a validated closed ended questionnaire.

*Root cause analysis:* Interview and brainstorming of healthcare workers for root-cause analysis was carried out as qualitative component. The healthcare workers providing direct care to the neonates were retrospectively interviewed using an open-ended questionnaire to find the causes leading to neonatal HAIs. Such interviews were conducted until the saturation of responses was met. Eighty different healthcare workers were interviewed to find the mechanism of spread of infection on open-ended questions in this process, until we reached to saturation of responses and similar responses were provided. The periodicity of responses were listed under six cause headings as per Ishikawa ^42^ i.e. A) Men, B) Material, C) Method/Process, D) Machine/Equipment, E) Environment, F) Policy.

The Ishikawa diagram, a diagrammatic representation of the root cause analysis was developed where the sources of infection were categorized under six domains. ^43^^,^^44^ Later it was recorded and tabulated to obtain descriptive statistics. Based on the periodicity of reporting of the contributor reporting was done through Pareto's chart. ^45^ Pareto's chart was able to highlight the significant contributors that cause HAIs among neonates. A graphical expression of the contributors to the existing problem highlighting the important ones to target was used. In Pareto’s chart, no categorisation was used, however, the contributors with <5% of reporting frequency were summarised together. The top 20% of contributors identified in Pareto’s chart were considered significant to neonatal HAIs. These contributors were taken up further as input to the development of the prevention bundle.

The average score and standard deviation table were developed on major contributors reported during brainstorming and survey. Experiences of healthcare workers were reported using the box plot due to outliers.

*Hospital Infection Control Committee’s (HICC) surveillance*: Surveillance reports were collected periodically from the air samples from NICU’s environment every three months of the year. The results of these samples were collected from the nurse in charge of NICU. There were two episodes of the suspected outbreak during the study when swab samples from the environment of a few of the neonates clinically acquiring HAIs were captured to identify the contributors. These swab samples were from the cradle side, surface swab, water sample, swab from feeding pallada, and equipment was collected directly by HICC members. The samples were analysed in the central microbiology lab and reports were submitted to NICU. As the official report arrived from a laboratory, the researcher noted the report's results and took it as probable contributors to neonatal HAIs. These contributors were considered critical to preventing HAIs and ensured improvisation through a review of literature for inclusion in the prevention bundle.

*System process mapping through observation:* The prospective regular random observations were done for the process and techniques used to provide the neonate care. The researcher followed procedural steps from the beginning until the end of the procedure and observations were marked against the checklist, prepared by the researcher, clinician and nursing staff of NICU, considering the standard steps to be followed during a procedure. These checklists were not given to the healthcare workers for marking. After the completion of the observations, the record used was entered in the checklist and on the Excel datasheet. The checklist based on the process are available under *Extended data.* ^90^

The time selected for observations was morning care time at 07:30 am; clinician’s clinical round time at 09:30 am and 3:00 pm; blood sample collection time at noon; invasive routine procedure time at 02:00 pm; medication/total parenteral nutrition preparation time at 03:30 pm; shift hand overtime at 07:30 pm. Various invasive and non-invasive procedures, care of the neonates and environment cleaning processes were focused during these random observations.

These observations were summarised in frequency and percentage under four categories in tabular format. The top 20% of frequent observations were considered as ‘scope for improvement’ for the prevention bundle development. ^46^

A survey on knowledge and practice assessment on hand hygiene practices among healthcare workers in NICU was also captured, analysed and reported, and is published in peer reviewed journal. ^47^ The observations on hand hygiene were classified under three categories i.e. a) time duration for hand hygiene practices, b) opportunities for hand hygiene before the procedures or activity and c) hand hygiene practice after the procedure or activity. Data collection forms for both survey and observation are available under *Extended data.* ^90^

*Failure effect mode analysis (FEMA):* FEMA was done on critical contributors identified through brainstorming, observation and system process mapping with the help of eight senior nurses and two clinicians.

We also performed FEMA on 21 identified processes that helped us in identifying the key steps that if missed or overlooked could lead to HAIs also it helped us to add and delete some of the steps that were carrying no value to the process. FEMA helped us to identify the critical key steps to be added to the process with more emphasis. FEMA was done on various steps in the care process that helped to find the critical 20% steps to focus on during the preparation of prevention care bundle however, many other activities scored <240 risk propensity number (RPN), and were not reported.

*Visit to benchmark NICUs in state*: Study visits to three different NICUs located in distant hospitals setting infection control policies were made. Observations, open ended interviews of healthcare workers and the study of infection control policies were recorded for each NICU. The findings were compared and presented to stakeholders at the study setting to find the best practices.

Based on the findings of all the observations, root cause analysis, process mapping, FEMA and observations at other NICUs the critical steps in the processes and the critical processes were identified. It was decided to develop standard care process for routine critical activities as a prevention bundle.

*Development of the prevention bundle:* We carried out three rounds of brainstorming meetings with clinicians, microbiologists and nursing staff to formulate a feasible prevention bundle. The presentations on root-cause analysis, observations, and processes were made available through email at least a week prior to the meeting and a presentation was delivered before the meeting.

Four clinicians were identified from the NICU including two senior (minimum 10 years of experience in NICU) and two junior clinicians (minimum 2 years of experience in NICU), they were also given an open-ended questionnaire with the developed process to identify the lacunae and improvise the process.

**Figure 1.**
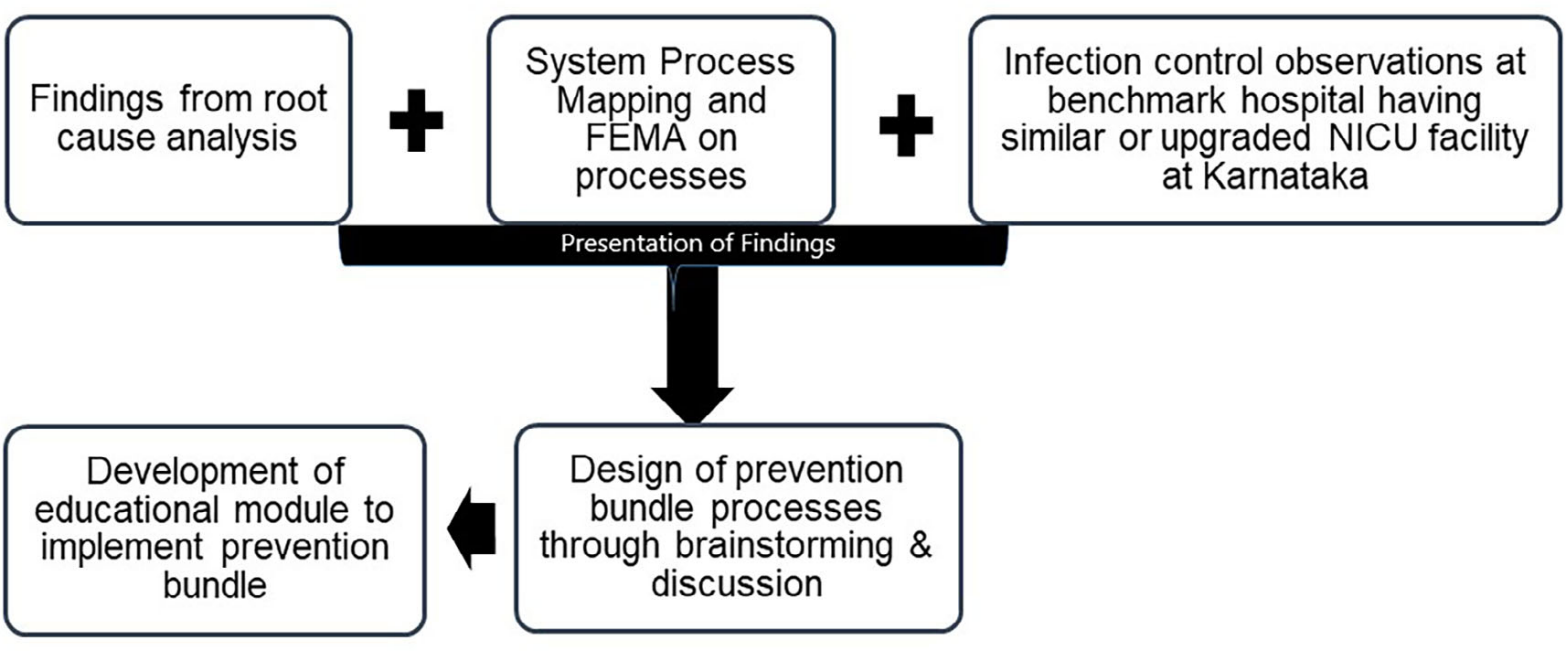
Process to draw inference for the development of prevention bundle.

Each meeting was of a minimum one-hour duration, clinicians were allowed to review each process for one week to provide their critical inputs. The inputs of all the healthcare workers involved in these activities were presented to experts’ i.e. senior neonatologist, senior microbiologist, three senior staff nurse and management representatives ( Figure 1). Their inputs were documented and were considered further for the design of the prevention bundle.

Each process had Input, Process and Outcome as suggested by Dr Donabedian. ^48^ Any surgical, equipment or manpower required to initiate and complete the process was considered under the input (mentioned within the process); the steps involved to carry out the task were arranged in sequential order in process, and the neonates benefiting were considered as an outcome of the process.

As the experts' suggestions were to combine the developed processes into two to three processes, the researcher finally prepared three processes after repeating four consultations with each clinician (four) and nurses (ten) that lasted for three months. The final three processes were identified as: A) Nursing care Process; B) Vascular access process and C) Cardiopulmonary resuscitation management process.

As per the experts' policy suggestions, do’s and don’ts and surveillance charts for NICU were also prepared in the supplement to the prevention bundle.

*Development of educational module to implement prevention bundle:* The developed three processes were further taken up for audio-video ^49^ and computer added presentation development. Three senior staff nurses were identified and trained for the process by the researcher. These nurses carried out all the steps of the process as per the instructions on a newborn mannequin in NICU on the cradle. Step by step each task was recorded for two procedure bundles: The nursing care process and the vascular access process. Audio-video recording was carried out during the day shift and three to four shots were taken for each task.

The raw video clips were edited in Windows Movie Maker ver 6.0 with background music obtained from licence free music library in YouTube. A due acknowledgement for the music source was given to the developer and source at the end of the video. The required audio clip was inserted and the length of the movie trimmed from 75 minutes to 15 minutes. Due acknowledgement was given to the video contributors. Final processing was completed in four months. A total of two videos, on the nursing care process and vascular access process was developed.

*Policy document:* This was prepared based on a review of the literature and the observations carried out at other healthcare facilities. The policy document included the incorporation of the initiative suggested during the brainstorming exercise and facilitated session. For the development of these policy documents, senior clinicians, a microbiologist, pharmacy expert and five nurses with a minimum of 5 years experience were involved. Prior to the discussion and meeting a draft policy was prepared which was communicated through email, feedback, suggestions and discussion until all agreed to the policy document. These policy documents can be found under *Extended data.* ^90^

*Do’s and Don’ts:* These rules were prepared based on the observations carried out at the study setting and other hospitals, review, ^50^^,^^51^ and the brainstorming exercise with healthcare professionals. Three interviews with the nurse manager and nurse in charge were carried out to make the changes according to the feasibility of carrying out tasks. Meeting and discussion with respiratory therapist staff were carried out to validate the content of the list. Final corrections, validation, and approval were taken from senior neonatology consultants, senior microbiologists, and their team before the study was adopted. The agreement of senior healthcare professionals that includes clinicians, nurses and HICC was sought. These rules were presented to senior nurses, clinicians and respiratory therapists for content validation. It took five rounds of changes during content validation. These documents can be found under *Extended data.* ^90^

*Surveillance charts:* The surveillance charts were developed based on the recommendations sought in the review of literature, the observations on practices at other hospitals and recommendations of the senior clinicians. These surveillance charts were presented for three rounds for content validation to nurses and clinicians.

Designing of assessment tools for pre and post-intervention: Ten multiple-choice and closed-ended questionnaires were developed for nurses and medical postgraduate students based on the identified processes and steps. Senior clinicians validated the questions (three) for their content validity and face validity. Internal consistency for the first set of questions was calculated with 15 samples of healthcare providers and cronbach's alpha was 0.812 for these questions.

At each intervention phase, the set of questions administered were different however the intent and purpose of those questions were the same only the language and phrases differ each time and internal consistency with cronbach's alpha was maintained above 0.75, where 15 healthcare providers helped in reviewing and answering these questions for validation. The respondents gave consent only at the beginning of the intervention, however, fifteen nurses were replaced by other staff at the middle of the intervention phase. The researcher included them from the next phase of intervention and took their written consent before the intervention. We anticipate the variation in the result due to these change of nurses in between the interventions. Still, no effort was made to adjust the findings for such variations and results were reported without any modifications considering the real time situations without any interventions.

The answers were evaluated for their correctness and a score of 1 was given to each correct answer whereas the wrong answer did not get any point. The summary of answer scores was prepared and reported as mean ± SD for each time the training was provided. A difference in mean and SD was computed to find the average change in pre and post-training. Students paired t-test with 95% CI at *p*-value fixed at <0.05 was considered a significant change in knowledge scores.

## Implementation of the prevention bundle and assessing its effectiveness

A quasi-experimental study design was adopted where three rounds of training to healthcare providers was provided to attain the behavioural modification and adoption to the design practices/process.

These experimental training were carried out in three training phases.

### Training phase I

In training phase I, the training was divided into two stages: implementation of the nursing care process bundle and implementation of vascular access process bundle along with cardiopulmonary resuscitation management process.

The first stage of the experiment began after finalizing the date and week with the nurse manager when all the nurses can be asked to be present to attend training. The stage was divided into two parts for the ease of implementation and considering optimal time for the training effectiveness to achieve. Daily post morning shift change at 02:00 pm all the nurses approximate 7-10 in numbers but occasionally 3-4 reaches to the training room.

One-hour video-based training was conducted for the consented participants. A pre-test was followed by a video show of the procedure. The researcher actively narrated and discussed each step. Concerns and queries of the participants were addressed instantly before continuing further. After active discussion, a post-test was conducted followed by due gratitude to their participation and request for their cooperation and support in the prevention of neonatal HAIs.

The second stage of training was given on the vascular access process to the healthcare providers. One-hour video-based training was conducted for the consented participants. A pre-test was followed by a video presentation of the procedure. The researcher actively narrated and discussed each step. Concerns and queries of the participants were addressed instantly before continuing further. After active discussion, a post-test was conducted followed by due gratitude to their participation and request for their cooperation and support in the prevention of neonatal HAIs.

Participants were also briefed on the cardiopulmonary resuscitation management process using the flow chart. Participants were briefed on the do's and don’ts. A post-test was conducted followed by due gratitude to their participation and request for their cooperation and support in the prevention of neonatal HAIs.

A total of 49 and 48 nurses were trained in both areas respectively. We excluded 5 and 6 nurses respectively as three nurses were part of the development of the training module and others were on long term leave of more than 30 days.

Training to five respiratory therapists was given on do's and don'ts and cardiopulmonary rehabilitation management process using a flow chart. For them also pre and post-training evaluation was carried out.

5 medical postgraduates were trained using role-play and simulation models along with video teaching aids only on vascular access process, cardiopulmonary rehabilitation process and do's and don'ts. The stimulation and role-play included sterile gowning and sterile gloves donning after surgical handwashing practices that were monitored. During monitoring of the healthcare providers, any deviation or wrong practice if found was corrected followed by carrying out the procedure again in a standard manner.

### Environmental changes

A review meeting was carried out with the hospital infection control committee (HICC) where the problem statement and its mitigation plan was discussed regarding preventing cross-infection and environmental changes. The review committee agreed to certain environmental changes which were:

Notice at shoe rack area to separate and place street shoe and NICU sleepers in respective racks; Prefilled hand sanitiser and notice to use it before entering to NICU premises. Separation of two different rack positions i.e. street footwear and NICU footwear, placement of hand sanitiser at the door inside to NICU to ease monitoring of hand hygiene practices.

Segregation and labelling of weighing machine as “Only for neonatal weight record” and “Use for diaper weight”. Removal of tissue paper roll for hand drying purpose. AMBU (Air Mask Bag Unit) bag replacement at every 72 hours of use.

Other changes that were requested but could not be implemented due to unavoidable factors were: Separate trolley for invasive procedures at Inborn and Out born area, the indent of fresh AMBU bags to replace AMBU bag every 72 hours.

### Training phase II

The training phase II was one stage and was on implementing the nursing care process bundle and the vascular access process bundle along with the cardiopulmonary resuscitation management process.

After finalizing the date and week with nurse manager when all the nurses can be asked to be present to attend training, the dates were fixed to the third week of December 2019. This time a little variation in the training approach was practised where the simulation stations were prepared and the participants were called to practice and show the learning. The corrections in practice were made at the simulation station only. There were five simulation stations, and these were:

a) Handwashing station
b) Medication preparation stations
c) Vascular catheter insertion station
d) Medication delivery station and
e) Neonatal routine care station

Each training time was generally scheduled from 1:00 pm to 02:30 and on two days was scheduled at 09:00 pm to 10:00 pm; two senior nurses were identified and sought for their help in implementing the training. These senior nurses had >10 years of experience in NICU and were also trained and briefed on their role at the station before beginning the training. Each participant was welcomed and gave a pre-test in written format. The consent was obtained only from those who were new to the training. After the pre-test, the participant was shown a video with oral narrations and descriptions by the researcher for 20 min duration. If any doubt arises in the middle of the video, it was discussed by pausing the video and then proceeded further. Comments, queries and discussions of the nurses were welcomed and encouraged during the presentation.

At each training day, three teams were formed among the participants. Post presentation the three groups were taken on three stations where the researcher and the senior nurse helper demonstrated the process and then assessed each participant while they demonstrated the practices. Correction in the practice if any deviation was found were made immediately at the station, and all the queries were resolved that were raised during a demonstration by the researcher. Each team practised and demonstrated their process at all the five stations. Participants were also briefed on cardiopulmonary resuscitation management process using the flow chart. Do’s and don’ts were briefed and handed over to each participant.

Post video and stimulation presentation each participant was given a written post-test to answer. The researcher thanked each participant and requested their cooperation and support in the prevention of the neonatal HAIs. In this stage, a total of 46 nurses were trained. We excluded eight nurses as three nurses were part of the development of the training module; three were utilized in training and others were on long term leave of more than 30 days.

Training for five respiratory therapists was given on Do’s and Don’ts and cardiopulmonary rehabilitation management process using a flow chart. For them, also pre and post-training evaluation was carried out.

The 5 medical postgraduates were trained using role-play and a stimulation model along with video teaching aids ^49^ only on vascular access process, cardiopulmonary rehabilitation process and Do’s and don’ts. The stimulation and role-play included sterile gowning and sterile gloves donning after surgical handwashing practices that were monitored. During monitoring of all the healthcare providers, any deviation or wrong practice was corrected there itself, followed by carrying out the procedure again in a standard manner.

### Training phase III

Phase III was one stage and was only on implementing the nursing care process bundle and the vascular access process bundle along with the cardiopulmonary resuscitation management process.

After finalising the date and week with the nurse manager, when all the nurses will be able to be present to attend training, the dates were fixed. This time a little variation in the training approach was practised; we identified seven nurse leaders with >5 years of experience, each nurse leader could choose any six nurses from their shift team to train further. The simulation stations were prepared, and the participants were called to practice and show the learning. The corrections in the practice were made at the simulation station only. There were two simulation stations and these were: Medication preparation stations; and vascular catheter insertion station.

The training time was scheduled as per the convenience of these senior nurses. The nurse assessed the handwashing, medication delivery, and neonatal routine care in real-time while providing the care to the neonate. The researcher trained each team leader and briefed on the training protocol with a pre and post-assessment questionnaire. Two to three staff nurses were welcomed to participant for pre-test and were trained by team leader each day completing their assigned six nurses in 3 to 6 days duration depending upon their shift duty. A video with oral narrations and explanation by the team lead for 10 minute duration was delivered. The nurses' comments, queries, and discussions were welcomed and encouraged during the presentation and resolved before moving further.

The team lead demonstrated the process and then assessed each participant while they demonstrated the practices at workstations. If found, corrections in the practice were made immediately at the station and the team leader resolved all the queries. Each team practised and demonstrated the process at two stations. Participants were also briefed on the cardiopulmonary resuscitation management process using the flow chart followed by do’s and don’ts in NICU.

Post video and stimulation presentation each participant was given a written post-test to answer. The team leader thanked each participant and requested their cooperation and support in the prevention of neonatal HAIs.

In this stage, 40 nurses were trained. We excluded 12 nurses as three nurses were part of the development of the training module, seven were utilized in training, and others were on long-term leave of more than 30 days.

Training to respiratory therapists and medical postgraduates could not be carried out at this phase due to the complete lock-down of the country owing to the surge of COVID-19 and as directed by the Institutional Ethics Committee. However, the prevalence of neonatal HAIs was captured until the completion of three months.

Categorical variables were reported using descriptive statistics, a time series graph was used for reporting changes in HAIs. Paired students T-test was performed on findings of the pre and post training.

### Ethical considerations

Written consent from all the participants of training were obtained before the training. They were informed that the data will be used for academic and research purposes. In no way would their personal information be disclosed or identity revealed either in the final report, publication or anywhere. Confidentiality will be maintained at all times, and privacy is respected. Institutional Ethics Committee (IEC) approval was taken, approval ID: MUEC/014/2016-17. CTRI (Clinical Trial Registry India) registration was done before starting the project, the confirmation ID was: CTRI/2017/08/009538.

## Results & discussion

### Root-cause analysis

Healthcare workers were interviewed on an open-ended written questionnaire, a total of 80 healthcare workers participated, and the majority of them were staff nurses with more than two years of experience (88%). Healthcare workers were identified as major contributors for HAIs ( Table 1).

**Table 1.** Scoring of healthcare workers on major contributors causing healthcare-associated infections.

Contributors	Mean score (max 5) *	SD
Healthcare workers	3.6	1.2
Equipment	2.9	1.0
Material	2.8	1.2
Policy/procedures *	-	-
Infrastructure *	-	-

* The score was based on the response rate and ranking of each variable by healthcare provides during root cause analysis where rank 1 = least contributor and rank order 5 = highly contributing to HAIs. *Skewed data, hence, mean +/- SD cannot be reported.

The ranking of the contributors to HAIs by these healthcare workers showed that prime contributors are healthcare workers (27%), followed by equipment (21%), material (21%), process/protocol (19%), and last is infrastructure (12%). Detailed root-cause analysis and multiple observations showed that non-compliance to handwashing practices, equipment disinfection/cleaning, IV line insertion, many invasive procedures, and improper aseptic techniques (>50%) are prime contributors causing HAIs among neonates.

**Figure 2.**
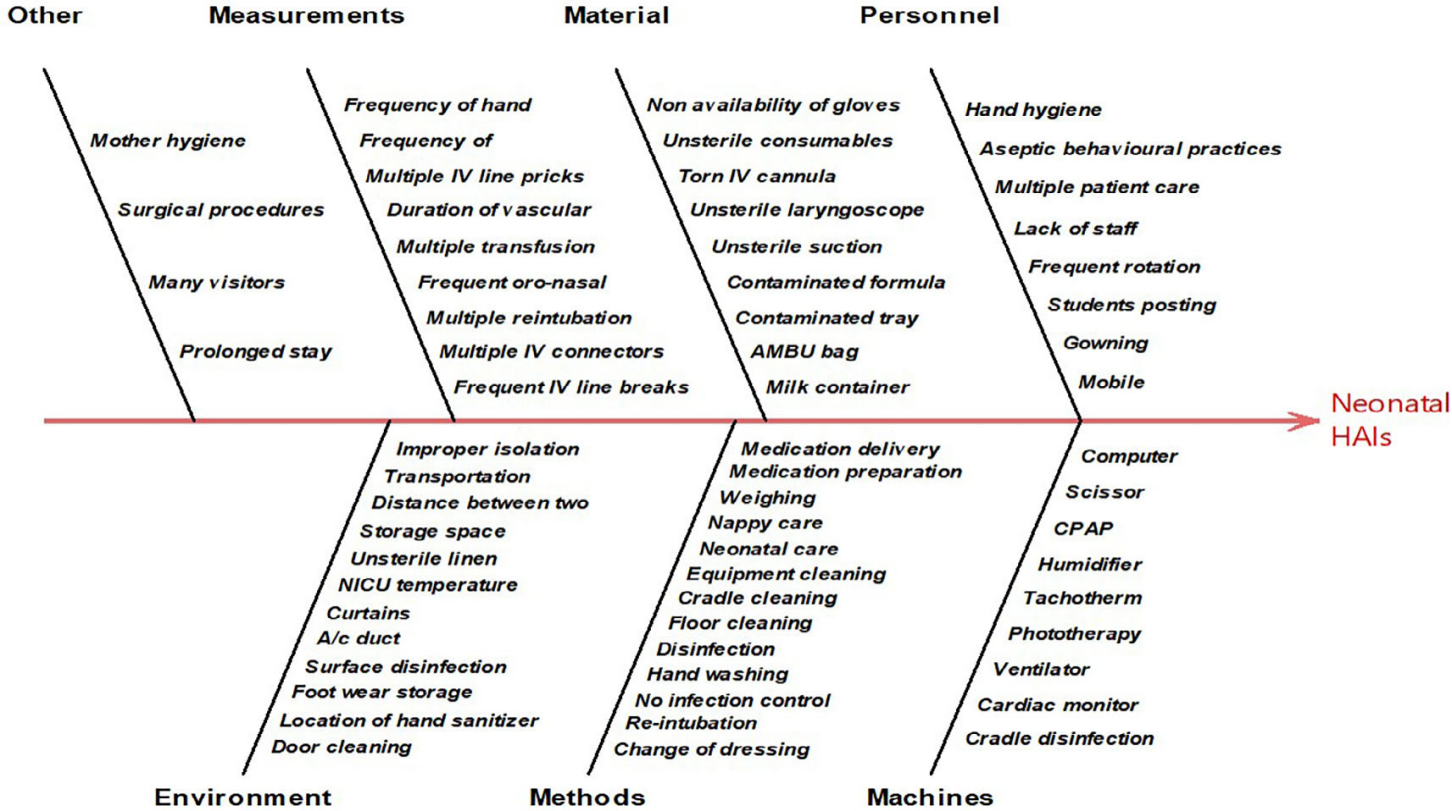
Observation and interview response in root-cause analysis.

**Figure 3.**
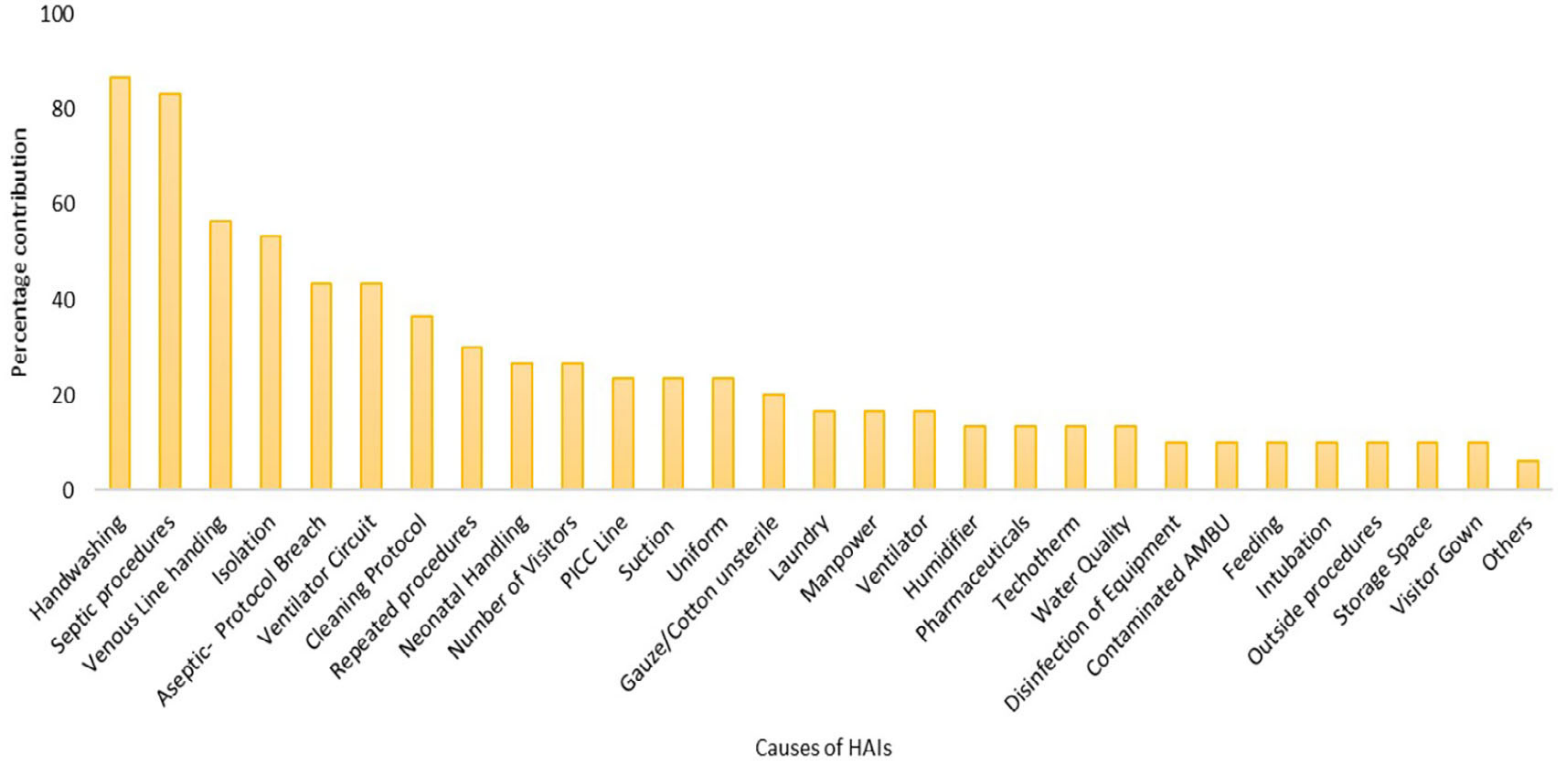
Percentage of contributors causing neonatal HAIs identified during root cause analysis by healthcare workers.

As the number and variation in responses of healthcare workers were very high the Ishikawa diagram was plotted to find any inference out of the responses ( Figure 2). To simplify further, the causes were ranked ordered on a scale of 1-5 by healthcare providers that helped for further analysis of responses in the form of Pareto’s chart ( Figure 3). As per the rank order the hand hygiene practices (87%), improper aseptic procedures (83%), improper handling of venous line (57%) and unable to provide timely isolation to sick/infected neonates (53%) were highlighted ( Figure 3).

***Observations against a checklist to find causes of HAIs in NICU***

Physical observations for 248 days and 1761 various infection control opportunities at the study site was made ( Table 2) and forty interviews on twenty-five variables were carried out for healthcare workers at NICU. The observations were carried out on the following practices: a) hand hygiene practices, b) care of intubated and ventilated patients, c) intubation process and ET (Endotracheal tube) suctioning, d) care of NIV (Non-invasive ventilation) patients (Oral suction and fixing of the nasal mask), e) insertion and care practice of PICC (Peripherally inserted central catheter) line, central line, umbilical line, arterial line and IV lines, f) practices on infant feeding g) cleaning and care practices at NICU, h) daily clinical rounds, I) medication preparation, sample collection, and blood transfusion practices.

**Table 2.** Critical observations to infection control practices in NICU.

S. No	Cause category (n) (%)	Sub cause	Cause description	Frequency of observations
1	Consumable (144) (9%)	Vascular line fixing tape	Aseptic storage and placement	76
		Storage	Medication vial, used nappy oil and a sterile gauze pad	62
		Stock out	Gloves	6
2	Environment cleaning (174) (10%)	Washbasin & tap handle	Daily disinfection	87
		Hand dryer switchboard	Daily disinfection	45
		Table to cut sterile gauze	Daily disinfection	42
3	Equipment (195) (11%)	Ventilator & other	Unused one kept near neonate without disinfection	96
		Scissor	Cleaning after use	83
		Handling by healthcare workers	An aseptic technique to handle	16
4	Healthcare worker (1196) (70%)	Physician	Hand hygiene before and after neonatal examination	228
		Allied health professional	Hand Washing	200
		Nursing	Hand hygiene before and after medication administration/feeding/in-between patient care and aseptic preparation of medication	180
		Nursing and physician	Hand drying	171
		Nursing	Inappropriate hub cleaning	168
		Nursing	Site cleaning & aseptic technique for GRBS	131
		Super specialist	Hand hygiene before and after examination	64
		All healthcare providers	Hand hygiene before and after touching Neonatal surrounding	54

### The hospital infection control committee (HICC) surveillance report

There were two occasions when HICC collected the environment sample for the identification of microorganisms. In its first occasion, only air samples were collected at different parts of the NICU that did not grow any microorganism on culture. On the second occasion, the swab samples were collected from various environments of the NICU as well as nasal swabs of healthcare workers.

A total of twelve swab samples, including one drinking water sample, were also sent to the microbiology lab for further analysis. There was a growth of *Klebsiella* sp. from the hand washbasin and neonatal cradle. The culture grew *Serratia marsescens* in swab culture of the oral cavity of one patient and feed container, *Klebsiella pneumoniae* in swab samples of cradle side, and no growth in a drinking watersample. There was no other growth of microorganisms on any of the other samples.

*System process mapping and FEMA on processes:* Multiple observations and interviews of healthcare providers were carried out to identify and understand the processes critical to infection control practices.

**Figure 4.**
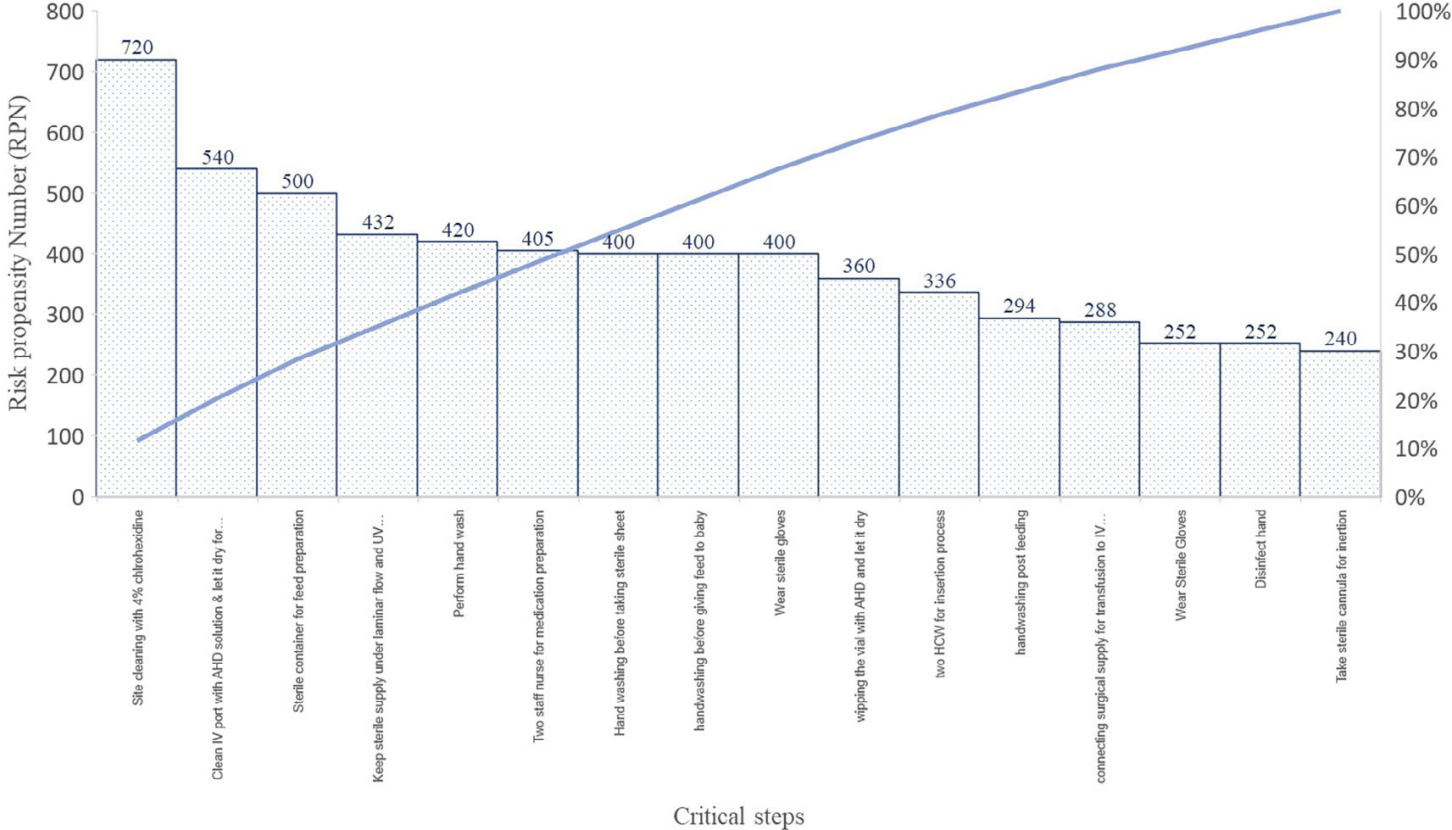
Pareto’s chart on RPN obtained during FEMA on critical steps of the prevention bundles.

We identified the 21 processes and its process validation with the help of healthcare providers at NICU. We found site cleaning with 4% chlorhexidine, disinfection of IV port before infusion, the sterile container for feed preparation, preparation of medication under laminar flow and strict hand hygiene practices were few critical steps of the processes ( Figure 4).

### Infection control observations at benchmark hospital having a similar or upgraded NICU facility at Karnataka

All the four NICU settings had an average of 80% occupancy and any two to three months per year, the occupancy goes up to 100%.

The observations were made and compared with existing practices in the study hospital ( Table 3). These observations were categorised further under the following headings:

**Table 3.** The infection control practices at different NICUs of Karnataka.

S. No.	Observations	Urban tertiary care teaching hospital #1	Urban tertiary care teaching hospital #2	Suburban tertiary care teaching hospital #1	Current setting
**Environment related practices**	**ICU divisions**	Intensive care Step down Isolation	Intensive care Step down Isolation	Inborn Outborn Stepdown Isolation	Inborn Outborn Stepdown Isolation
	**Number of beds**	30 goes up to 36	12 up to 22 separate cubicles	33 beds & three separate receiving beds and total goes up to 40	24 up to 28
	**Nurse to patient ratio Ventilated, Non Ventilated, Observation**	1:2 and 1:4 and 1:6	1:1; 1:2 and rarely 1:3	1:1; 1:2; 1:4	1:3 to 1:6
	**Curtains**	No	No	No	Yes
**Human related practices**	**Speciality consultants**	Restricted ophthalmology once a week	Very minimal entry on specific days	Very minimal entry on specific days	Restricted entry but poor hand hygiene practices
	**PG allocation**	One PG per cubicle area no interchange	No such practice	One PG per cubicle area no interchange	PG assigned for all the areas
	**Gown facility for Healthcare workers**	Need to remove all ornaments in hand and wear mandatory ICU Gown and mask (if necessary)	Mandatory to wear ICU gown	No Gown required	Gowns for mother only, customized NICU dress for nurses and PG students, no other gowning facility
	**Gown facility for Parents**	Need to remove all ornaments and wear ICU Gown and mask by all	Need to wear ICU gown	Need to wear a gown only by mother, not another family member	Available for mother only
**Practices/Process**	**Feeding practices Expressed breast milk (EBM)**	Feed preparation at the patient side	Feed preparation at the bedside	No such practices	No such practices
		EBM collection through sterile container only collected by the parent before every feed	EBM sterile feed container was given to the mother for feed preparation	feed preparation at a common point and any feed container is allowed	Feed preparation at a common point and any feed container are allowed.
	**Cleaning nappy area**	With sterile water plastic gloves	With sterile water Applying oil once per shift latex unsterile gloves	Cleaning with wet wipes not applying any oil occasionally wear latex gloves	Cleaning with Lukewarm sterile water followed by application of oil/Vaseline
	**IV-line insertion**	Sterile gloves The separate sterile kit of insertion baby gown to use	Sterile gloves with a sterile kit for insertion is used	Sterile gloves with no kit	Sterile gloves with no kit, sterile kidney tray are used with glove paper as drape
	**Random blood sugar check**	Plastic gloves and clean the glucometer with hand rub solution after each use	Latex gloves unsterile and clean the glucometer after every use	No gloves used, no disinfection of glucometer	No gloves are used, no disinfection of glucometer
	**Entry to NICU**	Street shoes outside room followed by clean slippers at a washing area complex	Shoe Cover to be worn over street wear	Street shoes outside room followed by clean slippers near to washing area	Change slippers at the entrance near the handwashing area.
	**The entry of New-born to NICU**	In Incubator/heated warmer only	In Incubator/heated warmer only	In hands/heated warmer/incubator, no set policy	In hands/heated warmer/incubator, no set policy
	**New-born screening**	Swabs from axilla and rectal are sent for MRSA screening	No such practices	No such practices	No such practices except sepsis screen for Outborn neonates
	**Non-invasive procedure**	Wear unsterile plastic gloves	Wear unsterile latex gloves	No gloves	No gloves
	**Laryngoscope**	Without bulb & handle send for autoclave	clean with 70% alcohol	clean with 70% alcohol	Washing with soap and water, rarely disinfection with 10% alcohol
	**Clubbing of invasive and non-invasive procedure together**	Yes	Yes	No	No
	**Normal saline for suction**	No	No	Occasionally	No
	**Transfusion preparation**	Once per day under laminar flow however currently non-functional	At bedside, no laminar flow	At bedside, no laminar flow	Under laminar flow, but adherence is poor
	**Change of cradle**	No such practice	Yes, every seven days complete change of cradle with the fresh cleaned one	No such practice	No such practice
	**Change of fluids/TPN**	Every 24 hours closed line	Every 48 hours only	Every 24 hours	Whenever fluid gets over
	**IV-line change policy**	every 72 hours	Every 72 hours	96 hours or SOS	SOS
	**Weighing machine**	Movable from one place to other. Disinfection pre and post-use	One place fixed. Use sterile paper no disinfection In between patient	Sterile paper is used, different weighing machines for different sections of the NICU.	One place fixed, used for both weighing neonate as well as a diaper. Use sterile paper no cleaning In between patient
	**Switching off the alarm**	With hand pre and post use of hand rub or patient-specific gown	With hand pre and post use of hand rub	With tissue paper or with hand, pre/post use of hand rub	No such practice, anyone switches off without hand hygiene
**Process monitoring**	**Handwashing before entry to ICU by all entrants**	Mandatory with CCTV monitoring and digital clock view to know the time for handwashing	Mandatory but no monitoring	Mandatory; nurses follow and confirm with each entrant to wash hands	No monitoring
	**Routine monitoring of the central line**	Each disconnection is recorded on a sheet	Each disconnection is recorded on a sheet	Each disconnection is recorded on a sheet	No recording of disconnection
	**Practice before touching neonate**	Must use hand rub for 20-30 sec. Wear neonate specific gown before handling or touching neonate	Washing hands with soap and water. No separate gown required	Use of hand sanitizer: not monitored, infrequent practices. No separate gown required	Handwashing followed by the use of hand sanitizer: not monitored, infrequent practices noted
	**Hand wash**	Mandatory and drying with sterile towel & hand rub	Mandatory and drying with tissue roll & hand rub	Occasionally however repeated hand rub solution was used as the solution was attached at the head side of the cradle	Occasionally followed; however, repeated hand rub solution was used as the solution was attached at the head side of the cradle.
	**VAP checklist**	Yes	Yes	Yes	No
	**CLABSI checklist**	Yes	Yes	Yes	No
	**IV line monitoring checklist**	No	No	Yes	No
	**Auditing period**	once per day	Once per day	No record	No set procedure, daily observations only
**Material/Supply related practices**	**BMW container**	Foot-operated separate	Foot-operated separate	Foot-operated common	Foot-operated common
	**Air-Mask Bag Unit (AMBU) bag**	After 7 days replace with a fresh sterile one	After 48 hours replaced with a sterile one	After 48 hours replace with sterile one or when soiled whichever is earlier	Same AMBU to continue till the stay of the baby, no sterilization in between
	**Patient files**	Plastic single-use	Plastic cover single-use	Board cleaned in between new patients	Board files not cleaned in between the patients
	**Stethoscope**	Single per patient	Single per patient	Single per patient; personal one is also cleaned with Sterillium	Single per patient
	**Heat Moisture Exchange (HME) filter**	No use	No use	Yes, changed every 48 hours	No use
	**Gown**	Sterile gown	Washed gown	No Gown required	Limited washed gown
	**Gloves**	Plastic gloves for unsterile process and latex for sterile process	latex gloves for both sterile and unsterile process	latex gloves for only during the sterile process	latex gloves for only during the sterile process
	**IV port**	One-way port	One-way port	Three-way stopper	Three-way stopper
	**Sterile pack for IV insertion**	Yes	Yes	No	No

Environment-related practices

Human related practices

Practices/processes

Process monitoring

Material/supply related practices

***Development of prevention bundle and training module***

Printed three bundle processes i) Vascular Access and care process ii) Nursing care and iii) cardiopulmonary resuscitation management process were developed. Audio-Video training & hands-on workstation training was also developed as prevention bundle module for healthcare workers to prevent neonatal healthcare association infections.

Two videos, a PowerPoint presentation, and five hands-on stations were developed as educational modules to implement the prevention bundle.

### To evaluate the effectiveness of the developed prevention bundle

**Figure 5.**
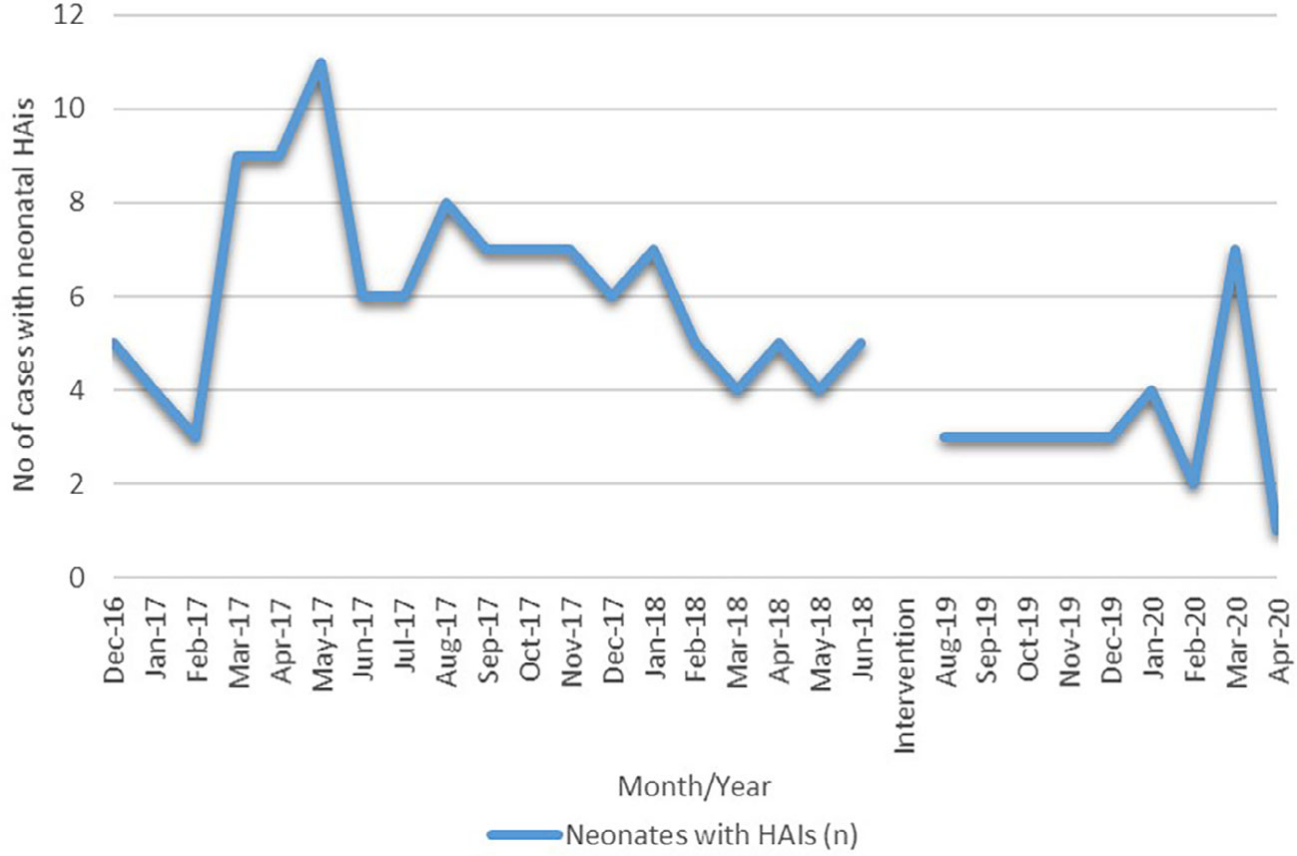
Time series cases of neonatal HAIs before and after the intervention.

There were four individual sets of training given to nursing staff and where there was a change of overall 21% knowledge score ( Table 4). Other healthcare workers were given training for only one time due to their unavailability and resistance to participating ( Table 5). Housekeeping workers were trained, but their knowledge assessment was not carried out. The prevalent microorganism identified during the interventional period was *Klebsiella pneumoniae* (41.3%), followed by *Acinetobacter baumanii* (27.5%). All these microorganisms were identified in blood samples. The rate of HAIs at the beginning of the intervention was 9.6, which at the end of the intervention was noted to be 7.0 as of April 2020 ( Figure 5).

**Table 4.** Knowledge scores of nursing staff on infection control practices pre and post-training.

Nursing training outcome							
S. no	Training title	Knowledge score pre-training		Knowledge score post-training		Change in %	Statistical significance
		Mean score (±SD)	% score	Mean score (±SD)	% score		
1	Nursing care process	4.7 (1.6)	46.5	8.4 (1.5)	84.1	37.6	p<0.0001 *
2	Vascular access process & CPR management	7.6 (1.2)	70.6	8.4 (1.3)	78.3	7.7	p<0.0001 *
3	Infection prevention bundle care	11.5 (1.8)	82.6	13.5 (0.6)	96.4	13.8	p<0.0001 *
4	Infection prevention bundle care	9.2 (1.3)	70.5	12.4 (0.6)	95.1	24.6	p<0.0001 *
Overall Average		8.3 (2.5)	67.6	10.7 (2.3)	88.5	20.9	P<0.0001 *

* Students paired t-test with CI 95% and level of significance <0.001.

**Table 5.** Knowledge scores of healthcare students on infection control practices pre and post-training.

Healthcare workers training outcome								
Training no	Profession	Training title	Knowledge score pre-training			Knowledge score post-training	% change	Statistical significance
			Mean (±SD)	% score	Mean score (±SD)	% score		
1	PGs (JR)	Infection prevention bundle care	10.6 (1.6)	81.5	13 (0)	100	18.5	p<0.05 *
2	Respiratory Therapist students (PG & interns)	Infection prevention bundle care	28.3 (0.83)	85.6	33 (0)	100	14.4	p<0.01 *

* Students paired t-test with CI 95% and level of significance <0.05.

At the beginning of the study, we aimed to bring at least a 5% change from the baseline rate of HAIs; the current interventional study was able to bring about a 26% reduction in the rate of HAIs reducing it from 9.6 to 7.0 per 100 admissions >48 hours. The bacteraemia rate fell from 5.2 per 1000 patient days to 2.6 per 1000 patient days and was statistically significant on two-tailed student t-test with 95% CI at *p*=<0.05 with *p*-value=0.00073.

The following method was used for the calculation:

$$\mathrm{ARR}=\left(\text{Control event rate}\right)-\left(\text{Experimental event rate}\right)$$

ARR=0.096−0.07=0.026

NNT=1/ARR=1/0.026=38.5

On average, 39 patients would have to receive this prevention bundle (instead of standard care) for one additional patient to prevent from HAIs.

## Discussion

This is one of the few studies that has attempted to bring holistic overview and approach to develop and analyze the prevention bundle to curb neonatal healthcare associated infections. The study used mixed-method approach to design the intervention and prevention bundle. There are variations in global infection control practices and resource constraints should be considered when developing local prevention bundles for neonatal healthcare-associated infections, indicating the need for further research in this area. ^52^

The current study used root-cause analysis is a retrospective approach for error analysis that investigates the direct or original error through qualitative approach that led to HAIs. ^53^^,^^54^ In the current study the prime contributors to HAIs among neonates, which were non-compliance to handwashing practices, equipment disinfection/cleaning, IV line insertion, many invasive procedures, and improper aseptic techniques. ^55^^,^^56^ Healthcare workers were identified as major contributors to HAIs similar to other published evidences. ^57^

As the number and frequency of observations and root cause analysis were highlighting improper hand hygiene practices & healthcare workers as one of the major contributors, reported by many, further investigation was carried out to find the areas of improvement. ^47^^,^^57^^,^^58^

Carrying out observations along with checklists and understanding the knowledge and perception of healthcare practices are followed routinely at NICUs. ^47^ Several previous studies have also identified healthcare workers as major contributors to HAIs, corroborating the findings of this study. ^59^ Few studies in different pediatric intensive care unit found that lapses in hand hygiene and adherence to aseptic practices were primary factors contributing to HAIs among neonates, where another study emphasized the importance of handwashing as the most important means of preventing nosocomial infection and suggested that each pediatric intensive care unit should develop programs to increase compliance with hand hygiene. ^60^^–^^62^ Several studies have identified the significance of equipment-related issues as contributors to healthcare-associated infections (HAIs), ^63^ a study in a neonatal intensive care unit and highlighted the significance of equipment-related issues as contributors to HAIs among neonates. ^64^^,^^65^ Their findings support the notion that inadequate equipment disinfection and cleaning can lead to infections, consistent with the results of the current study. ^66^ NeoCLEAN, a multimodal strategy to enhance environmental cleaning in a resource-limited neonatal unit, was found to be effective in reducing the incidence of HAIs. ^67^ This study suggests that proper environmental cleaning is crucial to prevent the spread of infections. It is important to note that inadequate equipment disinfection and or cleaning can lead to HAIs. Few surveillance based studies have reported that contaminated environmental surfaces and improper cleaning practices were the predominant contributors to HAIs, with healthcare worker-related factors playing a secondary role. ^65^ This discrepancy may be due to variations in healthcare settings, infection control practices, or population characteristics.

In many outbreak investigations, the swab samples grew microorganisms from the washbasin, cradle side and hands of the healthcare workers. ^68^^–^^71^ In our study on a swab or sample culture, we found growth of microorganisms on the washbasin, the oral cavity of the patient, sides of the cradle, and feed container. There was no growth on samples obtained from healthcare workers hands, water and air samples of the NICU. Performing periodic environmental surveillance may not necessarily contribute directly to infection control, but it can provide valuable insights into identifying areas where healthcare providers may require additional attention and retraining. ^72^ One has to be cautious especially in resource limited setting as environmental surveillance could add the cost burden. It is crucial to determine the primary objective when conducting either surveillance or root-cause analysis. Simply identifying and reporting on contaminated sources and subsequently disinfecting those specific areas may not necessarily result in a reduction of Healthcare-Associated Infections (HAIs). The underlying issue could potentially stem from incomplete disinfection procedures, the use of disinfection solutions that are over diluted compared to the recommended dosage, or training deficiencies among certain healthcare or support staff members. In the root-cause analysis, we discovered that one of the manufacturers of a particular incubator had recommended an overly diluted disinfection solution, which subsequently led all nurses to use the same diluted solution for complete environmental disinfection, however, identification and the correction in the dilution was implemented before the intervention phase.

Overall, the focus area for infection prevention and control is towards three area i.e. healthcare workers' hygiene practices (hand washing), ensure disinfectant and clean environment, and training of healthcare workers.

The Gemba walk ^73^^,^^74^ is a quality improvement tool that helps in better understanding the process and later standardizing the process. In our prevention bundle, this tool helped us in identifying eighteen processes and then later merging the essential ones and developing the standard three processes. Further improvement on these processes were based on the Donabedian model ^48^ in identifying the inputs to the process, the complete process as a whole and that lead to the desired outcome i.e. reduction in neonatal HAIs.

Three different multispecialty hospitals located in Karnataka were visited for observations on the prevention of neonatal HAIs.

Visiting neighboring benchmark hospitals could provide valuable insights and steps that can be considered while developing a prevention bundle, such as the development of checklists and policy protocols. A comparison of infection control practices in different hospitals could help identify best practices and areas for improvement. For example, a study comparing infection control practices in a Dutch and US hospital found that the infection risk scan (IRIS) is a tool that could be used in future research to measure the quality of infection control and antimicrobial use in a standardized way. ^75^

However, it is important to note that while infection control practices may be similar across different settings, there may be minor variations or modifications in existing infection control policies. ^76^^,^^77^

Additionally, some practices may add additional costs to patients, such as chlorhexidine baths for neonates before entry to the NICU. ^76^ Overall, visiting benchmark hospitals and comparing infection control practices can provide valuable insights for the development of effective prevention bundles.

Focus area of this study became blood stream infection associated with various procedures and general care of the neonate. We did develop the VAP prevention policy but there were only two cases in 18 months period with VAP and found that the practices followed at the current settings met the standard guidelines. ^78^ The standard guidelines for various specific types of infections or area to improve are already laid down e.g. environmental infection control in healthcare facilities, disinfection and sterilization in healthcare facilities by the CDC provide recommendations for cleaning and disinfecting environmental surfaces or the use of products by healthcare personnel in healthcare facilities. ^65^^,^^79^

We followed the various guidelines while preparing the prevention bundles a few of them are a) The guidelines for environmental infection control in healthcare facilities by the CDC provide recommendations for cleaning and disinfecting environmental surfaces in healthcare facilities ^65^; b) The guideline for disinfection and sterilization in healthcare facilities by the CDC discusses the use of products by healthcare personnel in healthcare settings such as hospitals, ambulatory care, and home care ^79^; c) A technical brief on environmental cleaning for the prevention of healthcare-associated infections prepared for the Agency for Healthcare Research and Quality provides evidence of the effectiveness of strategies for environmental cleaning and disinfection ^80^; and d) The Joint Commission's monograph on preventing central line-associated bloodstream infections emphasizes the importance of following evidence-based guidelines for the insertion and maintenance of CVCs to prevent CLABSI. ^79^ But the surveillance definition by CDC is for patients age <1 year, which underestimates the risks and physiology of the neonates who are more prone to HAIs compared to infant/child. ^81^^–^^83^

By developing the bundle care one cannot ensure that the infection will be prevented or controlled, this can be ensured with stakeholder's participation and its implementation by them. There are various approaches to provide training to healthcare workers, to ensure effective participatory delivery the study involved nurses in role play and audio-video recording to disseminate information. Training and re-training in a small frequent manner using multiple approaches helped for the behavioral change and developed further skills.

Training that is developed by involving the various stakeholders and discussion are found to be very effective than researcher-developed training alone. ^84^

Our study showed a reduction of 26% in neonatal HAIs, whereas other studies show a reduction up to 50% in neonatal HAIs after implementation of quality improvement initiatives among neonates <29 weeks across the neonatal network in Australia. ^23^ Developed countries have shown better outcomes after quality initiatives in NICU as compared with developing or resource-limited settings. ^14^ Few practices help to reduce BSI or CLABSI like the use of chlorhexidine coated IV cannula stopper ^85^ or single-use sterile tray for medication preparation and delivery, ^86^ cord care and neonatal bathing with chlorhexidine, ^87^^,^^88^ dedicated nurse leader for monitoring and training on infection control practices. ^89^ These practices help but do incur a cost burden on the healthcare organization and family of the neonate where out of pocket expenses on hospitalization drain out their entire savings. Not only the cost burden but also commitment from healthcare administration and change in policy for infection control practices is inevitable to implement such practices.

The strength of this study lies in two aspects i.e. process approach rather cause approach to prevent neonatal HAIs and stakeholder's involvement in development and implementation of the prevention bundle, as they reported, they owned the processes and implemented with dedication without any doubts on why this process is required to be followed.

We faced challenges while developing and implementing the bundle. Getting a commitment to participate and taking out extra time after their duty shift was a challenge. Limited staff availability during COVID-19 period was another challenge that led to a sudden surge in HAIs among surgical cases referred from other nearby hospitals.

Apart from above the study had many other limitations. The postgraduate medical and allied health students and interns were posted on a rotational basis for a duration ranged from 15 days to 3 months. This lead to variations in their infection control practices, if they missed the training session.

The current study setting was an open NICU where neonates from various specialization units like cardiology, gastroenterology, and orthopedic surgery were hospitalized and looked after by a specialty consultant. The training to these specialty consultants was not provided, that lead to varied infection control practices as each unit has medical students on a rotational basis also. The housekeeping staff also worked on a rotational basis and after every month the entire staff of housekeeping was changed. This was an uncontrolled factor for training to these staff and monitoring their infection control practices. We did not see many VAP cases hence the focus of the bundle was given to bloodstream infection and environmental disinfection including routine care to the neonates.

Future research can be carried out for development of such prevention bundles for various specialized intensive care units of the hospital. These prevention bundle may not be generalized and applicable to all the NICUs until discussed, modified and accepted by the nursing and clinical staff of the NICU.

## Conclusion

Root cause analysis revealed handwashing practices, inappropriate aseptic process, inappropriate disinfection of equipment and environment, inappropriate vascular line hub care, medication preparation and delivery and venous line handling were few important contributors to neonatal HAIs. We could identify eighteen important processes that were followed in NICU to deliver care to new-borns and were standardised as per the current setting. We found the vascular line hub care, handwashing practices and aseptic technique to be practised during procedures were key to prevent neonatal HAIs. Our developed prevention bundle was found to be effective and could bring down the infection rate. It had brought the behavioural change among the healthcare providers towards infection control practices and the audio-video aids along with hands-on workstations could continue as a training method for infection control practices to be adopted in NICU. Periodic training and developmental hands-on workshops would benefit the healthcare workers to follow behavioural change and practice infection control guidelines.

## Data Availability

Figshare: Development and evaluation of prevention bundle for neonatal healthcare-associated infections. https://doi.org/10.6084/m9.figshare.22284385.v7. ^90^ This project contains the following underlying data:
- Pre & Post training results.xlsx - FEMA Findings.xlsx - RCA findings.xlsx This project contains the following extended data:
- SQUIRE-2.0-checklist (1) filled.pdf - Blood product transfusion.docx - Central line.docx - ET suction.docx - Feeding.docx - Intubation.docx - IV line.docx - Medication preparation & delivery.docx - Oro-nasal suction.docx - PICC line.docx - TPN.docx - HAND HYGEINE_Survey – Copy.docx - Observer Checklist for Hand.docx - NICU basics for Infection Control Practices.docx - NICU CLABSI Prevention Bundle.docx - NICU VAP Prevention Bundle.docx - Consent form nurse.docx - Vascular access process Feb 2020 Pre.docx - Vascular access process Feb 2020 Post.docx - Nursing care process - pretest.docx Data are available under the terms of the Creative Commons Attribution 4.0 International license (CC-BY 4.0).
