# Supplementary material for: Development and evaluation of prevention bundle for neonatal healthcare-associated infections: an interventional study
Source: F1000Res. 2024 Jun 3;12:687. Originally published 2023 Jun 16. [Version 2] doi: 10.12688/f1000research.132819.2 (PMC12754361; doi:10.12688/f1000research.132819.2)
Supplement: Peer review history

# Reviewer report: Reviewer response for version 2

**DOI:** 10.5256/f1000research.163888.r438087

**Published:** 2025-12-30

**Authors:** Lizel Lloyd

**Affiliations:** 1 Stellenbosch University, Cape Town, South Africa

**Overall Assessment**

 The manuscript addresses an important issue—neonatal healthcare-associated infections (HAIs)—and proposes a locally feasible prevention bundle. While the study demonstrates innovation and practical relevance, several methodological, structural, and language issues need attention before publication.

**Major Issues and Suggestions**

**Abstract**

- **Grammar:** Ensure consistent past tense throughout (e.g., “was developed” instead of “is developed”).

**Introduction**

- **Paragraph 2:** Correct phrase “lowest motility rate” → should be “lowest mortality rate.”
- **Evidence:** Add a reference for the statement: *“The prevention bundle is available for adult population”* (Page 4).
- **CLABSI vs CRBSI:** Combine discussion; CRBSI is a subset of CLABSI, and prevention measures are identical—only diagnosis differs.
- **Repetition:** Remove redundant sentences:
  - Page 3: *“Since the increase in the amount of published literature related to the prevention of HAIs is increasing…”*
  - Page 4: *“There is an increase in the number of published literature related to the prevention of HAIs in the adult population…”* Suggestion: Merge into one concise statement.

**Methods**

- **Population:** Clearly define study population
- **References:** Page 4 cites reference 33 (1974, urethral catheterization in adults) for neonatal HAIs—replace with relevant neonatal source.
- **Ishikawa Reference:** Correct citation—currently uses 42; Ishikawa is 43,44.
- **Complexity:** Methodology is overly detailed and hard to follow. Suggest:
  - Use subheadings for clarity.
  - Summarize repetitive descriptions (e.g., training phases).
- **Specific Times:** Remove unnecessary time details unless scientifically justified.
- **Training Description:** Page 8—first and second stage descriptions are almost identical; combine for brevity.
- **Environmental Changes:** Present as bullet points for readability.
- **Terminology:** Use “scale” instead of “weighing machine.”
- **Overall:** Replace lengthy narrative with a summative approach.

**Results**

- **Figures:** Figure 2 and Figure 4 are blurry—replace with high-resolution versions.
- **Tables:**
  - Table 2 unclear—add explanatory caption.
  - Table 3 abbreviations (PG, SOS, BMW) must be defined.
  - Table 5 abbreviations (PG, JR) must be explained.
- **Disjointed Presentation:** Results feel fragmented; integrate findings logically.
- **Critical Question:** If healthcare workers are major contributors to HAIs, confirm whether **PPE access** was assessed—important in the LMIC context.

**Discussion**

- **Structure:** Avoid dual headings “Results & Discussion” and “Discussion”—merge into one section.
- **Grammar:**
  - Page 19, paragraph 2: Rewrite first two sentences for clarity, e.g.:
*“The current study used root-cause analysis, a retrospective approach to identify errors through qualitative methods that led to HAIs.”*
  - Second sentence:
*“The prime contributors to HAIs among neonates were non-compliance with handwashing, inadequate equipment disinfection, IV line insertion errors, invasive procedures, and improper aseptic techniques.”*
- **New Information:** The incubator disinfection issue (Page 20) was not mentioned earlier—introduce earlier or clarify why it appears here.
- **Incomplete Sentence:** Page 20 last sentence: Rewrite for clarity, e.g.:
*“CDC surveillance definitions for patients under 1 year underestimate neonatal risk compared to older infants.”*
- **Grammar Fixes:**
  - Page 21:
*“Training developed through stakeholder involvement is more effective than researcher-developed training alone.”*
  - Last sentence of paragraph 3:
*“Beyond cost burden, commitment from healthcare administration and policy changes are essential for implementing infection control practices.”*

**Conclusion**

- Grammar: Rewrite for clarity, e.g.:
*“The developed prevention bundle was effective in reducing infection rates and promoting behavioral change among healthcare providers. Audio-video aids and hands-on workstations can serve as sustainable training methods for NICU infection control.”*

**General Language and Grammar**

- Ensure consistent tense (past tense for completed actions).
- Avoid long, repetitive sentences; use concise academic phrasing.
- Correct awkward phrasing and improve readability throughout.

**Minor Issues**

- Abbreviation consistency across text and tables.
- Improve figure captions and table legends for clarity.
- Consider shortening introduction and methodology for better flow.

**Recommendations**

- Major structural and language revisions.
- Clarify population and sampling.
- Merge repetitive content and simplify methodology.
- Improve figures/tables and define abbreviations.
- Strengthen discussion with comparative analysis and evidence-based reasoning.

**Overall Recommendation**

**Accept with major revisions**

 The study is relevant and innovative but requires substantial methodological, structural, and language improvements for clarity, validity, and global applicability.

Is the work clearly and accurately presented and does it cite the current literature?

Partly

If applicable, is the statistical analysis and its interpretation appropriate?

Yes

Are all the source data underlying the results available to ensure full reproducibility?

Yes

Is the study design appropriate and is the work technically sound?

Yes

Are the conclusions drawn adequately supported by the results?

Yes

Are sufficient details of methods and analysis provided to allow replication by others?

Partly

Reviewer Expertise:

Neonatology with limited experience in infection prevention

I confirm that I have read this submission and believe that I have an appropriate level of expertise to confirm that it is of an acceptable scientific standard, however I have significant reservations, as outlined above.

# Reviewer report: Reviewer response for version 2

**DOI:** 10.5256/f1000research.163888.r285708

**Published:** 2024-09-06

**Authors:** Migbar Sibhat

**Affiliations:** 1 Pediatrics and Child health nursing, Dilla University, Dilla, Ethiopia

Thank you for allowing me to review the revised version of this manuscript. The authors tried to partly address the raised comments. However, there are pertinent issues that need to be considered yet before proceeding to final approval. Unless necessary revisions are made to the forwarded issues, the validity of the findings will be significantly affected. 

**Note:** Please don't bypass pertinent questions.

**Major issues:**

 1. Sampling: The authors responded that the major focus of the study was the qualitative part. However, it's mandatory to follow the methodological requirements for the quantitative part as far as the Mixed approach is applied. If the authors deem it insignificant, they can exclude the quantitative part. Otherwise, it is impossible to proceed as it is. 

 2. Population: The population of the study is not clearly described yet. Please address this issue before proceeding. 

 3. Interpretation issue: The authors respond as they prefer to apply the Localized terminologies over the internationally applicable terms. Since the finding is considered in an internationally peer-reviewed journal, the findings need to be replicable to the global scientific world rather than the local members. If mandatory the authors can operationalize so that the nurses in India can understand easily without overwhelming the scientific world.

 4. Regarding the years of experience, the authors did not touch on the point raised in the previous review round. Everybody knows that experience matters the level of performance and proficiency. The question is why to stick only to experience and ignore other criteria such as grades, number of training attended, and actual performance?

 5. Regarding the training issue, the authors' responses contradict what they have written in their main document. And some of the responses imply there was a methodological fallacy in the study. Inclusion without making sure that everyone is trained is completely unacceptable whether it is due to COVID-19 or whatever. I do have a major concern and the authors failed to address this in the required level of detail.

Is the work clearly and accurately presented and does it cite the current literature?

Partly

If applicable, is the statistical analysis and its interpretation appropriate?

Partly

Are all the source data underlying the results available to ensure full reproducibility?

Yes

Is the study design appropriate and is the work technically sound?

Yes

Are the conclusions drawn adequately supported by the results?

Yes

Are sufficient details of methods and analysis provided to allow replication by others?

Partly

Reviewer Expertise:

I have sufficient expertise to assess and critisize this work. I have specialized in pediatrics and neonatal care provisions. Hence, the prevention of neonatal and child mortality and morbidity is my primary area of expertise.  Furthermore, I also have portfolio of research publications and took part/engaged in different research activities such as grants, attending research conferences and workshops.

I confirm that I have read this submission and believe that I have an appropriate level of expertise to confirm that it is of an acceptable scientific standard, however I have significant reservations, as outlined above.

# Reviewer report: Reviewer response for version 1

**DOI:** 10.5256/f1000research.145771.r181523

**Published:** 2023-07-24

**Authors:** Migbar Sibhat

**Affiliations:** 1 Pediatrics and Child health nursing, Dilla University, Dilla, Ethiopia

The authors tried to address and develop a prevention bundle for HAIs in a single neonatal intensive care unit in India. Furthermore, the authors also tried to show the effectiveness of the prevention bundle in reducing the rate of neonatal HAIs and for healthcare workers’ training and development. However, the manuscript has several issues that need to be addressed, moderated, and clarified yet.

**Methods:**

- The methods section in general was too bulky and difficult to catch the main contents. Please try to make it specific and succinct, yet inclusive of the required contents.
- The population of the study was not well explained. Therefore, the population where the findings are going to be inferred (target/source population) needs to be clearly described in line with the population where the study is actually being conducted (study population) in the methods and materials section.
- For the quantitative part, the sample size required had to be scientifically estimated prior to the accomplishment of the study. In addition, appropriate sampling techniques should be applied rather than assigning samples/participants deliberately without scientifically supported methods of selection. Otherwise, the external validity of the study findings will be under question. The authors need to think over it with great emphasis.
- Under the methods section, you put a specific time schedule for some activities. “The time selected for observations was morning care time at 07:30 am; clinician’s clinical round time at 09:30 am and 3:00 pm; blood sample collection time at noon; invasive routine procedure time at 02:00 pm; medication/total parenteral nutrition preparation time at 03:30 pm; shift hand overtime at 07:30 pm.” Was there any scientific reason to put specific time to perform each procedure? What was the relevance of specifying the time for each activity?
- “These observations were summarised in frequency and percentage under four categories in tabular format. The top 20% of frequent observations were considered as ‘scope for improvement’ for the prevention bundle”. Why did you consider this cut-off point (20%)? Why not lower or higher than this cut-off point to declare the scope of improvement?
- Under Training Phase II, the application of the phrase “nursing care process bundle” did not seem exactly fit to the contents described below. Nursing care process is a broader term that is not limited to these procedures, but beyond. Hence, I recommend using the term “Nursing procedures” instead.
- Besides, “These senior nurses had >10 years of experience in NICU...” Did you think the year of experience will merely impact the level of proficiency? Why did you prefer years of experience over other criteria such as qualification, efficiency/actual performance, who took many pieces of training, and their grades?
- Pre-training performance of participants needs to be assessed to compare the effectiveness of the training. Despite the general HAIs estimations presented, I couldn’t see any statement regarding the performance of each specific procedure to be evaluated in the study Phases. This is the major concern that I appreciated in this manuscript. Otherwise, it could be difficult to accept the changes observed in this study without determining the baseline level of implementation for each specific procedure.

**Results:**

- “The ranking of the contributors to HAIs by these healthcare workers showed that prime contributors are healthcare workers (27%)”. How could you exclude if the inappropriate implementation of procedures by healthcare workers leading to HAIs was due to a lack of access to appropriate IPC and PPE-related infrastructures? It needs to be cautioned and clarified accordingly.

**Discussion:**

- Overall, the discussion seems a description of the procedures and methodologies rather than the discussion. Thus, please try to focus on discussing the pertinent findings based on your objective. Such details can be provided under the data management and processing sub-section of the “Methods” section, or probably as an introductory paragraph of the “Results” section.
- Moreover, almost all the findings and contents presented in the discussion section were not discussed as it has to be. The discussion mainly involves the interpretation of pertinent findings in a scientifically sound and clinically applicable manner, comparison with existing evidence, and provision of scientific or possible justifications/reasoning for the current findings and possible discrepancies with existing literature. Please put your justification and compare your findings with existing evidence in related literature. The whole discussion section requires thorough revision and re-writeup in these perspectives.
- “The nurses posted in NICU had rotational duty between NICU and Pediatric ICU, so after every 2-3 months there were 12 – 15 new nurses either due to rotation, leave, or attrition that needed to be trained for the infection control processes at NICU.” So, did you provide them training on entry, or included just without training? If training was provided, it should not be considered as a limitation. It can be a challenge rather. However, if you include them without training, that could be completely unacceptable. This can be considered as a methodological fallacy.  

Is the work clearly and accurately presented and does it cite the current literature?

Partly

If applicable, is the statistical analysis and its interpretation appropriate?

Partly

Are all the source data underlying the results available to ensure full reproducibility?

Yes

Is the study design appropriate and is the work technically sound?

Yes

Are the conclusions drawn adequately supported by the results?

Yes

Are sufficient details of methods and analysis provided to allow replication by others?

Partly

Reviewer Expertise:

I have sufficient expertise to assess and critisize this work. I have specialized in pediatrics and neonatal care provisions. Hence, the prevention of neonatal and child mortality and morbidity is my primary area of expertise.  Furthermore, I also have portfolio of research publications and took part/engaged in different research activities such as grants, attending research conferences and workshops.

I confirm that I have read this submission and believe that I have an appropriate level of expertise to confirm that it is of an acceptable scientific standard, however I have significant reservations, as outlined above.

# Response

**Published:** 2024-03-15

**Authors:** Usha Rani

**Affiliations:** Manipal University, India

RESPONSE TO QUERIES ON METHODOLOGY

- The population of the study was not well explained. Therefore, the population from which the findings are going to be inferred (the target /source population) needs to be clearly described in line with the population from which the study is actually being conducted (study population) in the methods and materials section.

For observations, interviews, root-cause analysis, and system process mapping, the population was all the healthcare workers providing direct or indirect care to neonates hospitalized in the level IV Neonatal Intensive Care Unit (NICU) of a tertiary care teaching hospital. However, to explore the best practices of peer healthcare facilities, observations were made in another three different level IV NICU's of Karnataka where one was located in Semi-Urban district and two were in Urban metro city.

- For the quantitative part, the sample size required had to be scientifically estimated prior to the accomplishment of the study. In addition, appropriate sampling techniques should be applied rather than assigning samples/participants deliberately without scientifically supported methods of selection. Otherwise, the external validity of the study findings will be under question. The authors need to think over it with great emphasis.

The current study is more focused on qualitative data synthesis. Qualitative studies requires interviews till the saturation of responses reaches or maximum 30 participants. For the current study more than 30 interviews were done and made focus group discussion of 6 members each. Interviews were carried out on all the healthcare workers working in the study site NICU and brainstorming was carried out on each process with minimum 5 nurses & two paediatrician for each identified processes. The observations were carried out for a year on all the identified processes critical to spread of healthcare associated infections. We conducted observations considering minimum 30 observations per process or till saturation of observations arrived.

  

- Under the methods section, you put a specific time schedule for some activities. “The time selected for observations was morning care time at 07:30 am; clinician’s clinical round time at 09:30 am and 3:00 pm; blood sample collection time at noon; invasive routine procedure time at 02:00 pm; medication/total parenteral nutrition preparation time at 03:30 pm; shift hand overtime at 07:30 pm.” Was there any scientific reason to put specific time to perform each procedure? What was the relevance of specifying the time for each activity?

Some of the elective routine procedures are done just after the next shift change of nurses like baby bath, changing the linen, doing suctioning, cleaning the equipment, and routine blood collection for investigations carried out electively at 07:30 am. We specified the time for each activity in a study ensuring that data is collected during specific periods of the day when certain procedures are more likely to occur this timing could provide a more accurate picture of the processes carried out in the ICU. These procedures other than the emergency situations, are routinely carried out on specific time of the day and hence we have specified the time.

- “These observations were summarised in frequency and percentage under four categories in tabular format. The top 20% of frequent observations were considered as ‘scope for improvement’ for the prevention bundle”. Why did you consider this cut-off point (20%)? Why not lower or higher than this cut-off point to declare the scope of improvement?

Pareto's principle, also known as the 80/20 rule, was introduced by the Italian economist Vilfredo Pareto [1], states that roughly 80% of effects come from 20% of causes in 1896 in regards to economics. Later it was reintroduced to quality improvement by Joseph M. Juran in 1950 . The top 80% of frequent observations were considered as 'scope for improvement' for the prevention bundle lead by top 20% of frequent causes, the study could prioritize the most significant issues that need improvement. This approach helped ensuring that the most significant issues are addressed first, which have the greatest impact on developing the prevention bundle. We could also use resources efficiently by applying this principle.

- Under Training Phase II, the application of the phrase “nursing care process bundle” did not seem exactly fit to the contents described below. Nursing care process is a broader term that is not limited to these procedures, but beyond. Hence, I recommend using the term “Nursing procedures” instead

We thank and appreciate the reviewer for the suggestion, in India nurses frequently use the phrase "Nursing care" rather "Nursing procedures" in order to more contextualize and personalized to develop acceptance among nurse the phrase was used and same terminology was used in the prevention bundle which is being sent for Indian Copyrights office hence, we apologies that we are not able to change the terminology at this stage.

- Besides, “These senior nurses had >10 years of experience in NICU...” Did you think the year of experience will merely impact the level of proficiency? Why did you prefer years of experience over other criteria such as qualification, efficiency/actual performance, who took many pieces of training, and their grades? All the nurses working in NICU had similar qualification, we did considered efficiency as per the hospital policy efficient nurses and most trained nurses were given a role of team leader and or shift Incharge. These nurses were involved while implementing the prevention bundle and all had more than 10 years of experience and were senior rank holder. There were literature evidences to involve senior nurses along with other nurses, however we tried including even junior nurses who had <2year of experience too but they were involved only in the development of the prevention bundle.

Senior nurses with more years of experience have more skill-based knowledge, which could be promising in implementation of a prevention bundle and removing the acceptance barrier. They were familiar with the NICU environment, including the equipment, procedures, and protocols and were able to differentiate the existing policy and procedures from the proposed. Previous research has shown that years of experience can impact the level of proficiency of nurses in the NICU[2]. Therefore, it is consistent with previous research to consider years of experience when developing and implementing a prevention bundle [3, 4]. We hope that this answers the above query .

- Pre-training performance of participants needs to be assessed to compare the effectiveness of the training. Despite the general HAIs estimations presented, I couldn’t see any statement regarding the performance of each specific procedure to be evaluated in the study Phases. This is the major concern that I appreciated in this manuscript. Otherwise, it could be difficult to accept the changes observed in this study without determining the baseline level of implementation for each specific procedure.

Pre-training performance of participants was assessed through observation that was part of objective 1, under table 2 and figure 2 & 3; later due to feasibility issues and COVID-19 restrictions only knowledge assessment was carried out through questionnaires, which is reported in Table 4 along with incidence and rate of HAIs during implementation phase.

RESPONSE TO QUESTIONS ON RESULTS

- “The ranking of the contributors to HAIs by these healthcare workers showed that prime contributors are healthcare workers (27%)”. How could you exclude if the inappropriate implementation of procedures by healthcare workers leading to HAIs was due to a lack of access to appropriate IPC and PPE-related infrastructures? It needs to be cautioned and clarified accordingly."

Thank you for identifying and highlighting this important concern. Although we have not mentioned in the results but there was continuous complete supply of PPE to healthcare facility and since the hospital is accredited by Indian National Regulatory Body, all the policy documents and protocols were in reach of all the healthcare providers of NICU. We haven't ignored this part rather this was assessed and ensured before determining that the prime contributor was healthcare workers and not the disruption of supply through observations and hospital supply records.

- Overall, the discussion seems a description of the procedures and methodologies rather than the discussion. Thus, please try to focus on discussing the pertinent findings based on your objective. Such details can be provided under the data management and processing sub-section of the “Methods” section, or probably as an introductory paragraph of the “Results” section. Moreover, almost all the findings and contents presented in the discussion section were not discussed as it has to be. The discussion mainly involves the interpretation of pertinent findings in a scientifically sound and clinically applicable manner, comparison with existing evidence, and provision of scientific or possible justifications/reasoning for the current findings and possible discrepancies with existing literature. Please put your justification and compare your findings with existing evidence in related literature. The whole discussion section requires thorough revision and re-writeup in these perspectives.

We have rewritten the discussion as suggested by the reviewer.

- “The nurses posted in NICU had rotational duty between NICU and Pediatric ICU, so after every 2-3 months there were 12 – 15 new nurses either due to rotation, leave, or attrition that needed to be trained for the infection control processes at NICU.” So, did you provide them training on entry, or included just without training? If training was provided, it should not be considered as a limitation. It can be a challenge rather. However, if you include them without training, that could be completely unacceptable. This can be considered as a methodological fallacy.

All the nurses were trained as soon as they join the NICU and retrained for those on rotation duty, or leave every month under the study period and after the study training is persistent every three months.. As the training module had written and audio-video recording along with a senior staff mentor it was easy for us to ensure that each one is trained when they start practicing in the NICU. However, during March 2020 due to COVID-19 restrictions and very limited number of nursing staff on duty, it was not possible to verify that the training is imparted to the nursing staff who would have missed the training due to various reasons.

# Reviewer report: Reviewer response for version 1

**DOI:** 10.5256/f1000research.145771.r181526

**Published:** 2023-07-18

**Authors:** Ronald L. Castelino

**Affiliations:** 1 Faculty of Medicine and Health, University of Sydney, Sydney, NSW, Australia

Thank you for the opportunity to review this very interesting manuscript. Overall, the article is very well written and addresses a critical healthcare issue and provides valuable insights into the prevention of neonatal healthcare-associated infections.

 Below are some suggestions for consideration.

- The introduction is quite lengthy. Many of the interventions are described in detail. These can be summarised and included in the discussion for comparison purposes. Or a summary of the evidence of these in reducing HAIs and mortality should be included.
- Second paragraph in the introduction is confusing. I suggest deleting the lowest and highest ranges provided.
- Significant grammar edits are needed. In many paragraphs present tense is used instead of past tense. E *.g. the concept of bundles was developed...*
- Paragraph 7: What is cluster care?
- What are some of the local stats? What is the current rate of HAI in the local setting and mortality rates? This should be added to the introduction.
- What were the specific objectives?
- Who were the 80 health care workers interviewed? I suggest providing a breakdown. Experience level in NICU etc.
- Who developed the prevention bundles? Who developed the training? Who interviewed the health care workers?
- Who categorised/matched the domains? Who tabulated/transcribed the interviews?
- The main objective listed in the manuscript includes - *The current study was carried out to develop and evaluate prevention bundles for neonatal healthcare-associated infections (HAIs).*
- Methods stating hand hygiene practices - how does this relate to the current objective? These aspects are not clear and not linked.
- Who provided the training in phase 1? What were their qualifications?
- Why was the training implemented in 3 phases? Please justify.
- Have there been any outcomes differences between health care acquired infection vs acquired in the community?
- Can you comment on the generalisability of the intervention and sustainability?

Overall, even though it tackles an important topic the manuscript would benefit from an English edit and more importantly can be made more succinct.

Is the work clearly and accurately presented and does it cite the current literature?

Yes

If applicable, is the statistical analysis and its interpretation appropriate?

Yes

Are all the source data underlying the results available to ensure full reproducibility?

Yes

Is the study design appropriate and is the work technically sound?

Yes

Are the conclusions drawn adequately supported by the results?

Yes

Are sufficient details of methods and analysis provided to allow replication by others?

Yes

Reviewer Expertise:

Pharmacy

I confirm that I have read this submission and believe that I have an appropriate level of expertise to confirm that it is of an acceptable scientific standard.
